# Pinworm (*Enterobius vermicularis*) in children and adults: a practice-oriented narrative review and household–school algorithm

**DOI:** 10.3389/fpubh.2026.1780558

**Published:** 2026-05-25

**Authors:** Osama Albasheer, Gassem Gohal, Ali Al-Makramani, Mai Mustafa, Amani Abdelmola, Suhaila Ali, Fatma Ayish, Afnan Madkhali, Afaf Hakami, Hatim Alessa, Mohammed Muqri, Tahani Madkhali, Doaa Ayish, Ai Yahya Maashi, Elham Shabanh

**Affiliations:** 1 Department of Family and Community Medicine, Jazan University, Jazan, Saudi Arabia; 2 Department of Pediatric Medicine, Faculty of Medicine, Jazan University, Jazan, Saudi Arabia; 3 Department of Internal Medicine, College of Medicine, Jazan University, Jazan, Saudi Arabia; 4 Department of Family Medicine, Jazan University Hospital, Jazan University, Jazan, Saudi Arabia; 5 Department of Internal Medicine, Jazan Military Hospital, Jazan, Saudi Arabia; 6 Jazan Ministry of Health, Jazan, Saudi Arabia

**Keywords:** children, household, hygiene, pinworm infection, review, tape tests

## Abstract

Enterobiasis (pinworm infection), caused by the nematode *Enterobius vermicularis*, remains among the most common helminth infections worldwide and disproportionately affects children and caregivers in households and institutional settings. Despite its high prevalence, practical guidance is often fragmented across primary care, pediatrics, and public-health sources. We conducted a SANRA-guided, clinician-oriented narrative review synthesizing evidence on the epidemiology and psychosocial burden of enterobiasis, transmission dynamics in households and educational settings, best available diagnostic and therapeutic strategies (including recurrent disease), and context-specific considerations relevant to the Gulf region. We included 27 sources published between January 2015 and December 2025. Across the literature, three consecutive early-morning perianal tape tests consistently outperformed stool ova-and-parasite examinations. Standard two-dose therapy administered 14 days apart (mebendazole, albendazole, or pyrantel pamoate) achieved high cure and egg-negative rates when paired with synchronized household treatment and a right-sized hygiene bundle (morning shower before dressing, nail care, targeted laundering, and hand hygiene coaching). Routine school exclusion is generally unnecessary when treatment has started and the child is otherwise well. Guidance for special populations supports hygiene-first approaches in early pregnancy, with selective pharmacotherapy after the first trimester when needed. For persistent or recurrent symptoms, adherence verification and household coverage remain the first steps; extended (“pulse”) regimens may be considered in selected cases, although evidence is based mainly on pragmatic experience and expert consensus rather than randomized trials. The reported association with appendicitis remains inconsistent and should be interpreted cautiously. Overall, family-centered strategies—diagnosis using tape tests, completion of the Day-14 repeat dose, synchronized household treatment, and feasible hygiene—can reduce reinfection cycles and caregiver distress.

## 1 Introduction

Pinworm (threadworm), caused by *Enterobius vermicularis*, remains one of the most frequent helminth infections of childhood and a persistent nuisance in households and schools (1). Transmission is primarily fecal–oral via hands and fomites, with clustering in children aged 3–10 years and recurrent spread to siblings, caregivers, and staff in congregate settings (2). While the medical course is usually mild, the day-to-day burden is tangible: disturbed sleep, irritability, enuresis, and anxiety for families; repeated clinic or pharmacy visits; and periodic classroom notifications (1, 3). Reinfection is common when management is piecemeal or unsynchronized across household members (3).

Despite this everyday relevance, guidance is fragmented across pediatric texts, primary-care resources, and public-health advisories. Recommendations on when to test versus treat, how to coordinate dosing across the household, what hygiene is realistically necessary, and how schools should respond are not consistently integrated for frontline use (4). Evidence in special circumstances—pregnancy, infants, institutional clusters—can be scattered, and regional data from Gulf settings remain limited (1, 3–5). In short, clinicians and families often receive advice that is either overly generic or impractically burdensome, contributing to ongoing cycles of reinfection.

Despite several intestinal parasite surveys in Saudi Arabia and neighboring Gulf states (6–8), contemporary, community-based surveillance specifically targeting *E. vermicularis* using standardized perianal tape testing is limited. Most regional studies have relied on stool microscopy or mixed parasite panels, methods known to underestimate pinworm prevalence, and few have sampled school-aged children using optimal diagnostic techniques (7, 9).

This narrative review addresses that gap. Our objectives are to (i) summarize the recent evidence base on burden, transmission, diagnosis, and treatment of *E. vermicularis*; (ii) clarify areas of consensus and uncertainty relevant to primary and community care; (iii) align clinical recommendations with public-health and school policies; and (iv) highlight regional considerations for the Gulf/Saudi context. The aim is to provide a clear, practice-oriented synthesis that can reduce reinfection, minimize disruption for families and schools, and support consistent counseling in busy clinics.

## 2 Methods

This article is a clinician-oriented narrative review guided by SANRA (Scale for the Assessment of Narrative Review Articles) principles (10). Our purpose was to consolidate practical, up-to-date guidance for primary care, pediatrics, and school/public-health teams on diagnosis, household/school control, and recurrence prevention of *E. vermicularis*.

We undertook a targeted, non-exhaustive search of PubMed/MEDLINE, Embase, Scopus, Web of Science, and Cumulative Index to Nursing and Allied Health Literature (CINAHL), together with authoritative public-health guidance [e.g., Centers for Disease Control and Prevention (CDC), the UK Health Security Agency (UKHSA) and National Health Service (NHS), and the World Health Organization Regional Office for the Eastern Mediterranean (WHO/EMRO), and relevant national/state health agency websites], focusing on literature from January 2015 to December 2025 and the final search was performed on 31 December 2025. We included sources published in English; studies in other languages were excluded. Search terms were applied iteratively and included combinations of ‘pinworm’, ‘threadworm’, ‘*Enterobius vermicularis*’, ‘enterobiasis’, ‘diagnosis’, ‘tape test’, ‘treatment’, ‘recurrence’, ‘household transmission’, and ‘school’.

Selection was purposive: sources were included when they directly informed real-world decisions in primary/community settings (e.g., when to test vs. treat, two-dose regimens with Day-14 repeat dosing, synchronized household treatment, pragmatic hygiene measures, and school attendance). We excluded sources without clear relevance to diagnosis, treatment, prevention of recurrence, or implementation in community settings. We reviewed the last decade’s literature through targeted database and guideline-source searches and included 27 sources most relevant to the objectives of this narrative synthesis, comprising primary studies, systematic reviews/meta-analyses, and guidelines/clinical overviews.

Primary studies contributed context on burden and implementation challenges; systematic reviews/meta-analyses supported core epidemiologic and diagnostic points; and guidelines/clinical overviews informed practice-ready recommendations aligned with public-health policy. In synthesizing the evidence, greater weight was given to systematic reviews/meta-analyses and international guidelines for core recommendations, while primary studies were used to provide contextual insights on epidemiology and implementation. Where discrepancies existed, conclusions were guided by consistency across higher-level evidence and alignment with public-health guidance.

Evidence was organized into thematic domains (burden/psychosocial aspects; household/school transmission; diagnostics; therapy & recurrence; special populations; regional Gulf/Saudi notes; and common controversies). Within each theme, we favored convergent, actionable messages consistent across multiple reputable sources and explicitly noted uncertainties where evidence was limited.

Throughout the manuscript, evidence levels were classified into three pragmatic categories to support transparent interpretation.

High-certainty evidence refers to findings supported by systematic reviews and/or meta-analyses, and/or consistent recommendations across multiple international guidelines (e.g., CDC, WHO, NHS/UKHSA).

Moderate observational evidence refers to findings derived from multiple observational studies (e.g., cohort, cross-sectional, or case series) demonstrating consistent direction of effect but lacking randomized or pooled quantitative synthesis.

Expert-consensus guidance refers to recommendations based primarily on authoritative clinical guidelines, narrative reviews, or expert opinion where direct comparative or high-level empirical evidence is limited or absent.

These categories are used pragmatically to reflect the strength and consistency of available evidence and do not represent formal GRADE assessment.

Assignment of evidence levels was based on the type of source, consistency of findings across sources, and direct relevance to clinical decision-making, with preference given to higher-level evidence where available.

## 3 Discussions/observations

### 3.1 Global epidemiology and psychosocial burden

#### 3.1.1 Global prevalence and patterns

Modern pooled analyses estimate a meaningful ongoing burden of enterobiasis in children globally, with notable geographic variability (1, 11, 12). Prevalence is influenced by crowding, sanitation, and hand-hygiene behaviors typical of early schooling years (3). Outbreaks in childcare centers and dormitories reflect the ease of fomite-mediated spread and frequent re-infection (5).

#### 3.1.2 Clinical and psychosocial impact

Pinworm transmission is closely linked to everyday household and classroom routines (13). Following nocturnal oviposition in the perianal mucosa, eggs become infectious within hours and can persist on skin, clothing, bedding, and nearby surfaces for days, facilitating rapid spread through hand-to-mouth behavior (3). Night-time itching leads to scratching and contamination of fingernails, with eggs readily transferred to hands, toys, and frequently touched surfaces. Thumb-sucking, nail-biting, and snacking further sustain the fecal–oral cycle, particularly in young children (3, 14, 15). Routine practices such as dressing before bathing and shaking bed linens may amplify exposure by transferring or dispersing eggs into the immediate environment (3).

Beyond physical discomfort, recurrent enterobiasis can impose a meaningful psychosocial burden on children and families (16). Nocturnal pruritus disrupts sleep and may contribute to daytime fatigue, irritability, reduced concentration, and poorer school functioning. Caregivers often report sleep disruption, anxiety about recurrence, and confusion about whether repeated symptoms reflect treatment failure or reinfection. At school, visible scratching or discomfort may lead to embarrassment and stigma, which can affect self-esteem and social participation. The need for repeated household-level treatment cycles can further increase stress and impact family quality of life.

Reinfection is commonly driven by asynchronous household management, particularly when only the symptomatic child is treated while siblings or caregivers remain untreated (17). Autoinfection may persist when nail-biting, long nails, or inconsistent hand hygiene continues after dosing (15). While pets are not reservoirs, short-term contamination of sleepwear, bedding, and shared towels may contribute to continued transmission when hygiene measures are inconsistently applied around treatment days (3, 13, 17).

In schools and childcare settings, shared play spaces and routine post-toileting eating behaviors can facilitate clustering, particularly if only symptomatic children are treated or return-to-school guidance is unclear (3).

Overall, effective control depends on synchronized treatment, simple and feasible morning routines (shower and clean underwear), nail care, consistent hand hygiene, and targeted laundering at treatment initiation, while avoiding burdensome or unrealistic “deep-cleaning” expectations (3, 13).

### 3.2 Diagnosis

#### 3.2.1 Clinical suspicion

Classic features include nocturnal perianal pruritus, sleep disturbance, and occasionally visible small white worms near the anus or in stool (3). Vulvovaginitis or urethral irritation may occur in females. Abdominal pain and irritability are non-specific (17, 18).

#### 3.2.2 The perianal “tape test”

The diagnostic test of choice is the early-morning perianal adhesive tape test performed before bathing or toileting (4, 19). Three consecutive early-morning perianal tape tests consistently outperform stool examinations for the diagnosis of *E. vermicularis*, a finding supported by high-certainty evidence from systematic reviews and convergent international guidance (20, 21). In contrast, routine stool ova-and-parasite examinations have low diagnostic yield, as eggs are deposited perianally rather than shed reliably in stool. Tape tests can be collected at home with clear instructions (press sticky side to perianal skin; apply to slide or store in a sealed bag; repeat for three mornings).

Testing is useful in atypical presentations, persistent symptoms after correctly administered therapy, or institutional outbreaks (21). In high-probability cases (classic symptoms plus household exposure), with ready access to safe and effective therapy, empiric treatment is reasonable (Table 1), provided that education and follow-up are arranged (3).

**Table 1:** Diagnostic testing versus empiric treatment in suspected enterobiasis.

Clinical scenario	Recommended approach	Evidence basis
Classic nocturnal perianal pruritus with household exposure	Empiric two-dose therapy + synchronized household treatment	High-certainty evidence; guideline consensus
Visible worms reported by caregiver	Empiric treatment	Moderate observational evidence
Recurrent symptoms after treatment	Perianal tape test ×3 mornings	High-certainty diagnostic evidence
Atypical symptoms (abdominal pain, vulvovaginitis, diagnostic uncertainty)	Diagnostic testing before treatment	Expert-consensus guidance
Institutional outbreak (school/nursery)	Coordinated testing and/or mass treatment	Public-health guidance

### 3.3 Treatment

#### 3.3.1 First-line anthelmintics

For uncomplicated *E. vermicularis*, contemporary guidance converges on a simple principle: give a single therapeutic dose and repeat it on Day 14 (3). The first dose clears susceptible adult worms, and the second covers larvae emerging from residual eggs once they complete the short prepatent period. Standard therapy is highly effective, with reported cure/egg-negative rates commonly around 90–95% in clinical overviews, while some trial summaries report rates in the ~93–100% range when assessed around 2 weeks’ post-treatment; however, reinfection is frequent, particularly when household contacts are not treated simultaneously (22). In practice, three agents are used interchangeably with high clinical success when the schedule is respected and reinfection is prevented. Mebendazole is typically given as 100 mg orally once and repeated 2 weeks later; pyrantel pamoate is dosed at 11 mg/kg (to a maximum of 1 g) with the same two-week repetition and, in many settings, is available over the counter; albendazole is administered as 400 mg orally once on an empty stomach and repeated on Day 14 (3). Differences in apparent cure or egg-negative rates across agents are generally small and are more often explained by implementation factors, including under-dosing in young children, omission of the second dose, and asynchronous household management.

The choice among these drugs is therefore pragmatic and guided by availability, cost, local approvals, formulation preferences, and individual contraindications (4). Adverse effects after a single dose are usually mild and transient (e.g., abdominal discomfort, nausea, and headache), and serious events are rare (3, 4). Symptomatic itch often improves within a few nights; a bland barrier cream can help with excoriation, while topical corticosteroids should be reserved for clear eczematous secondary dermatitis rather than routine use.

#### 3.3.2 Synchronize treatment across the household

Treating the index case and all household members simultaneously, regardless of symptoms, is the single most impactful intervention for preventing reinfection, supported by moderate observational evidence and strong expert-consensus guidance across public-health agencies and clinical reviews (3, 11).

#### 3.3.3 Recurrent or persistent enterobiasis

Recurrence clusters in young children, large or crowded households, and nursery/school settings where hand-to-mouth behaviors are common (3). Observational series consistently show that households treating only the index child rebound within weeks, whereas synchronized dosing lowers repeat positives. If symptoms persist beyond 2–3 weeks after the Day-14 dose, first check adherence and household coverage (4, 23, 24). If uncertainty remains, do three early-morning perianal tape tests on consecutive days before bathing/toileting. Three samples meaningfully increase sensitivity (stool ova and parasite examination [O&P] is poor for pinworm). Also consider mimics that prolong itch despite cure: perianal dermatitis/eczema, group A streptococcal perianal infection, irritant dermatitis, hemorrhoids, or thread-like lint misidentified as worms. In recurrent or persistent enterobiasis, extended or “pulse” regimens (e.g., repeated dosing every 14 days over several cycles) are based on expert-consensus and pragmatic observational evidence rather than randomized trials. Available data suggest that benefit is driven primarily by improved adherence and reinforced hygiene practices rather than pharmacologic escalation alone.

#### 3.3.4 Environmental and household context

Beyond treatment synchronization and hygiene adherence, broader environmental conditions may influence exposure to parasitic infections. While the available evidence is not specific to *E. vermicularis*, environmental health studies have suggested associations between factors such as indoor dampness, humidity, and increased risk of gastrointestinal and parasitic infections in children (25, 26). Future studies should explore whether environmental factors influence reinfection risk or adherence to control strategies in *E. vermicularis* infection.

#### 3.3.5 Special populations

In early pregnancy, recommendations for hygiene-only management are derived from expert-consensus guidance and precautionary principles, reflecting limited direct safety data rather than evidence of harm. When symptoms are significant, post-first-trimester mebendazole use is supported by moderate observational safety data and longstanding clinical experience (27, 28). Breastfeeding is generally compatible with mebendazole; albendazole is minimally excreted in milk. Local regulatory guidance should be followed (3).

Infants/toddlers: Safety data are limited under age 2 years; weigh risks and benefits and involve pediatric specialists (24). Emphasize household hygiene and caregiver treatment to reduce exposure (28).

Institutional settings (schools, childcare, long-term care): Coordinate with public-health teams. Mass, simultaneous treatment repeated in 2 weeks may be appropriate in outbreaks (3).

School, nursery, and work: Exclusion is usually not required if the child feels well and treatment/hygiene measures are underway. Inform schools so environmental and hand-hygiene messages can be reinforced.

### 3.4 Controversies and complications

#### 3.4.1 Appendicitis

The association between *E. vermicularis* and appendicitis remains inconsistent and is supported by low-to-moderate observational evidence with substantial heterogeneity across studies (29). Much of the literature consists of retrospective appendectomy and pathology series, which are vulnerable to selection bias because they include only surgically treated patients rather than all individuals presenting with abdominal pain or suspected appendicitis. In addition, studies use variable denominators (e.g., all appendectomies vs. histologically confirmed appendicitis), inconsistent histopathologic definitions (incidental luminal presence vs. inflammatory involvement), and often provide limited clinical correlation, all of which restrict causal inference. Reported frequencies therefore vary across settings, but are typically low (commonly <1–3% of appendectomy specimens in modern pediatric cohorts) (30). Overall, current data do not establish *E. vermicularis* as a causal trigger of appendicitis. Clinically, suspected appendicitis should continue to be managed according to standard diagnostic and surgical pathways, while identification of *E. vermicularis* (e.g., in pathology specimens) should prompt consideration of concurrent enterobiasis management where clinically appropriate, including patient and household treatment to reduce reinfection and onward transmission (31).

#### 3.4.2 Genitourinary tract involvement

Occasional migration to the vulva/vagina may cause vulvovaginitis, discharge, or dysuria in girls; treatment of enterobiasis plus local care usually resolves symptoms (17). Rare ectopic presentations have been described, but severe complications are exceptional.

### 3.5 Regional lens: gulf and Saudi Arabia

The core diagnostic and management principles for *E. vermicularis*, including early-morning perianal tape testing, two-dose anthelmintic therapy administered 14 days apart, and synchronized household treatment, are grounded in the parasite’s life cycle and transmission biology and are therefore broadly applicable across settings, including the Gulf region and Saudi Arabia (11).

Evidence specific to *E. vermicularis* in Saudi Arabia is limited and, in some instances, dated. Much of the available local literature reports on intestinal parasitic infections (IPIs) as a broader category, rather than pinworm alone, and estimates vary by region, school setting, and population characteristics such as migrant status (6). Earlier pediatric surveys described *E. vermicularis* prevalence in the low single digits among schoolchildren, while some settings, particularly rural or lower-income communities, showed higher overall IPI burdens. More recent hospital-based analyses continue to document IPIs, with declining prevalence reported in some urban centers (32).

Importantly, because IPI surveillance methods are heterogeneous and stool-based screening may under-detect *E. vermicularis* relative to consecutive tape-test protocols, these findings should be interpreted as contextual indicators rather than precise estimates of pinworm burden.

In the absence of robust contemporary, pinworm-specific prevalence data, several implementation factors are nevertheless relevant for control in Gulf settings. Larger or multigenerational households may increase the number of close contacts and complicate synchronized same-day treatment when counseling is delivered only to a single caregiver. Shared caregiving responsibilities (including parents, grandparents, and domestic workers) may further influence adherence, making clear written instructions and explicit role allocation (e.g., who receives treatment, who supervises the Day-14 repeat dose, and who implements hygiene measures) particularly important.

Healthcare-seeking patterns may also affect recurrence. In some contexts, families may initially seek advice from community pharmacies for nocturnal itching, enabling rapid access to treatment but potentially increasing the risk of incomplete household coverage, missed repeat dosing, or limited counseling on reinfection prevention if care is symptom-driven. Conversely, strong primary care access and established school health systems in parts of the Gulf may support consistent implementation when clinicians emphasize that recurrence typically reflects reinfection rather than treatment failure and provide simple, standardized household instructions.

Taken together, the proposed household–school algorithm may be especially useful in Gulf practice as a pragmatic implementation framework, but it should be adapted to local routines, medication access, and caregiving structures. Future Saudi and Gulf research should prioritize pinworm-specific school-based studies using consecutive early-morning tape-test protocols, alongside implementation research examining household clustering, adherence barriers (including Day-14 completion), care pathways (clinic vs. pharmacy-first), and acceptability of standardized counseling tools. Such evidence would strengthen regional guidance, improve burden estimates, and inform context-specific public-health planning.

### 3.6 A practical household algorithm (clinician-facing)

**Figure 1:**
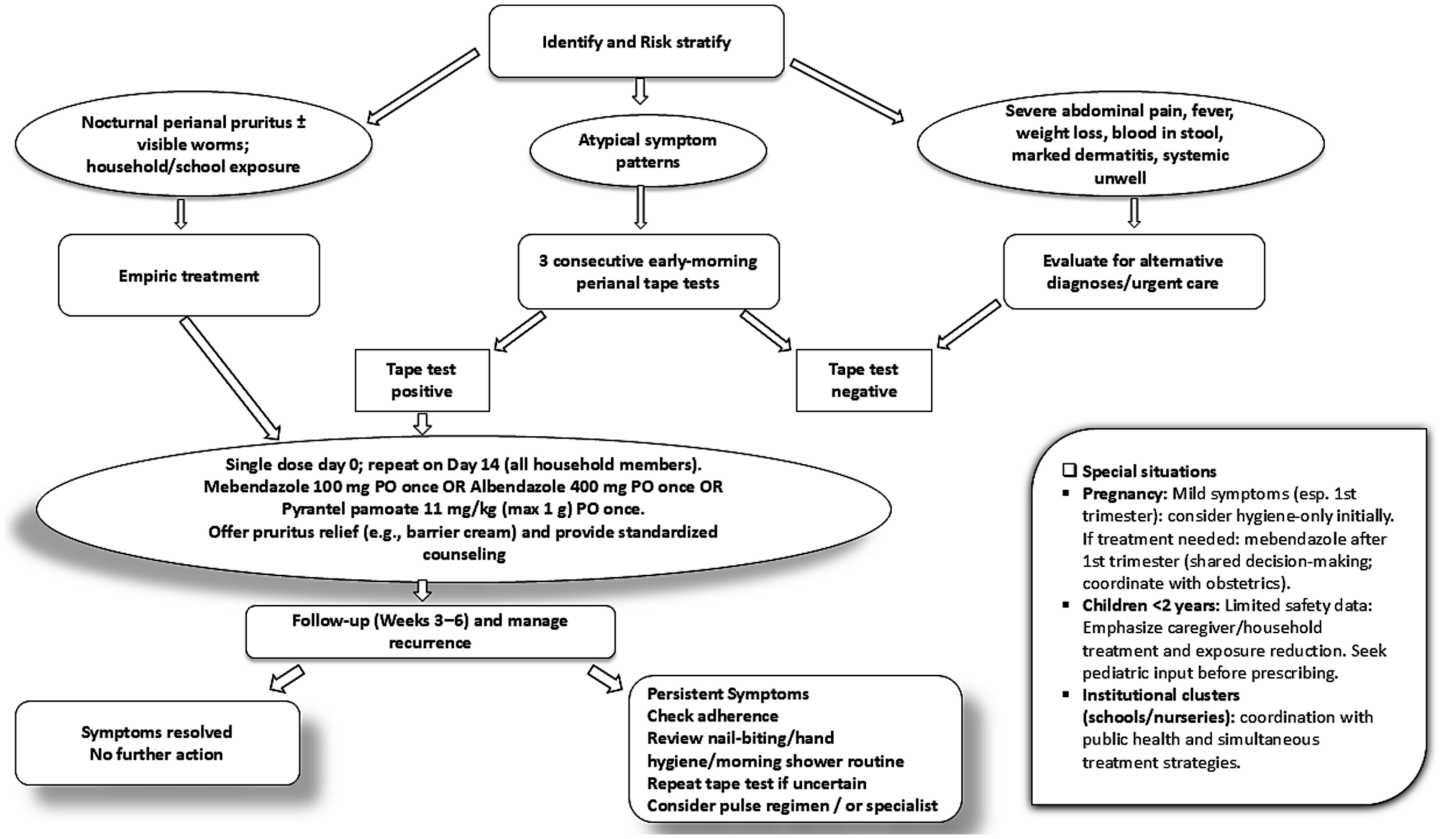
Practical household management algorithm for enterobiasis: Stepwise household-centered algorithm for the diagnosis, treatment, and prevention of recurrent *E. vermicularis* infection. The flowchart emphasizes risk stratification, appropriate use of perianal tape testing, synchronized household treatment, pragmatic hygiene measures, and structured follow-up to minimize reinfection.

To translate the available evidence into a usable framework for frontline care, we propose a stepwise, household-centered management algorithm for *E. vermicularis* infection. The algorithm integrates risk stratification, appropriate use of diagnostic testing, synchronized household treatment, and pragmatic hygiene measures, with structured follow-up to minimize reinfection and unnecessary disruption to families and schools. This clinician-facing approach emphasizes implementation factors, particularly simultaneous treatment of household contacts and adherence to a two-dose regimen, over drug selection alone. The proposed pathway is summarized in Figure 1.

#### 3.6.1 Step 1: Identify and risk-stratify

Patients with suspected *E. vermicularis* commonly present with nocturnal perianal pruritus, household clustering, or visible small worms. Atypical symptoms or red flags (e.g., severe abdominal pain, fever, weight loss, or blood in stool) should prompt evaluation for alternative diagnoses.

#### 3.6.2 Step 2: Decide on testing versus empiric therapy

Where diagnostic uncertainty exists, clinicians should recommend three consecutive early-morning perianal tape tests performed before bathing or toileting. In high pre-test probability cases (classic symptoms with household exposure) and ready access to safe, effective therapy, empiric treatment is reasonable, provided that education and follow-up are arranged.

#### 3.6.3 Step 3: Treat on Day 0 and provide standardized counseling

A single dose of an appropriate anthelmintic should be administered on Day 0 (e.g., mebendazole 100 mg once, albendazole 400 mg once, or pyrantel pamoate 11 mg/kg once, according to age/availability). The index case and all household members should be treated on the same day, regardless of symptoms. Symptomatic relief (e.g., barrier creams for pruritus) may be offered. A pragmatic hygiene bundle should be advised for the first week after dosing: morning shower before dressing, clean underwear daily, nail trimming with avoidance of nail-biting, routine handwashing after toileting and before meals, and targeted laundering of underwear/pajamas/bed linens, while avoiding shaking linens and unnecessary “deep cleaning.”

#### 3.6.4 Step 4: Repeat treatment on Day 14

All treated individuals should receive the same agent and dose again on Day 14. Hygiene measures should be reinforced for one additional week around the second dose.

#### 3.6.5 Step 5: Follow-up (Weeks 3–6) and manage recurrence

If symptoms resolve, routine retesting is not required. If symptoms persist or recur, the first step is to verify adherence (including synchronized household dosing and completion of the Day 14 dose) and review ongoing behaviors that sustain reinfection (e.g., nail-biting and inconsistent morning hygiene). Where uncertainty remains, repeat three early-morning tape tests. For recurrent or persistent cases, extended (“pulse”) regimens and/or specialist input may be considered, noting that such approaches are supported mainly by expert consensus and pragmatic observational evidence rather than randomized trials.

#### 3.6.6 Step 6: Special situations

In pregnancy, particularly in the first trimester, hygiene-only approaches can be considered initially when symptoms are mild; if treatment is necessary, mebendazole after the first trimester is commonly used following risk–benefit discussion and coordination with obstetric care.

In infants under 2 years, limited safety data favor emphasizing caregiver/household treatment and exposure reduction, with pediatric input before prescribing.

For institutional clusters (schools/nurseries), coordination with public health is recommended, and simultaneous treatment strategies may be considered depending on local policy and outbreak context.

### 3.7 Caregiver education script (for clinicians to adapt)

The above algorithm and the following caregiver instruction tool represent an expert synthesis of the available evidence and clinical guidance intended to support practical implementation in primary care; they have not been formally validated as clinical decision-support tools.

To support consistent and practical caregiver counseling, we provide a concise, clinician-adaptable instruction tool (Box 1) for use in primary care and school health settings.

Box 1 **Caregiver instructions for pinworm (threadworm).**

- Pinworm is common and treatable.
- Give the medicine TODAY (Day 0) and repeat the same dose in 14 days (Day 14).
- Treat all household members on the same day, even if asymptomatic.
- For 7 days after each dose:

◦ Morning shower before dressing
◦ Clean underwear daily
◦ Trim nails; discourage nail-biting/thumb-sucking
◦ Wash hands after toilet and before meals
◦ Wash pajamas/underwear/bed sheets at the start of treatment; avoid shaking linens.

- No deep cleaning is needed—routine cleaning is enough.
- Children can usually attend school/nursery if they feel well and treatment has started.
- Contact your clinician if itching persists after the second dose or symptoms recur.

Adapt advice for pregnancy and infants <2 years as directed by a clinician.

#### 3.7.1 Recommendations and future directions

Future work should prioritize practical research and quality-improvement questions that directly strengthen real-world control of *E. vermicularis* in primary care and school settings. Key priorities include evaluating implementation packages that improve completion of synchronized household treatment and the Day-14 repeat dose; developing and testing behavioral approaches to reduce nail-biting and thumb-sucking in early childhood; and assessing school-linked education or communication strategies that improve acceptability and minimize stigma. Diagnostic research should focus on the feasibility of parent-collected tape tests for wider use in community and school programs. Evidence gaps in special populations warrant prospective observational cohorts or safety registries to support counseling in pregnancy and in children under 2 years. Finally, contemporary Gulf-region studies are needed, particularly school-based prevalence surveys using standardized consecutive early-morning tape-test protocols, ideally incorporating household clustering and reinfection dynamics to inform locally tailored implementation.

### 3.8 Limitations

Evidence quality is uneven, with relatively few randomized trials and substantial reliance on observational data and expert guidance, particularly for recurrent disease and special populations. The restriction to English-language publications may have introduced language bias and limited the inclusion of relevant studies published in other languages. Regional data from the Gulf are also limited. Accordingly, the strength of conclusions is not uniform: core recommendations grounded in the life cycle and transmission biology of *E. vermicularis* (e.g., early-morning tape testing, two-dose therapy 14 days apart, and synchronized household treatment) are supported by consistent public-health guidance and broad clinical consensus, whereas some aspects of recurrent/persistent infection management (including extended or “pulse” regimens) rely more on pragmatic observational evidence and expert consensus than on comparative trials. Nevertheless, convergent recommendations across public-health agencies and clinical reviews support the proposed algorithm as a practical, clinician-oriented framework.

Generalizability also warrants caution. The proposed household–school algorithm is intended for primary care, pediatrics, and community/public-health settings, but its implementation may require adaptation in healthcare systems with different medication availability, diagnostic access, school health infrastructure, follow-up capacity, and caregiving arrangements. Therefore, the algorithm should be applied as a pragmatic framework rather than a rigid protocol, with local adaptation to regulatory, resource, and sociocultural contexts.

The absence of contemporary, tape-test–based prevalence data from Saudi Arabia limits precise regional burden estimates and highlights the need for targeted school-based epidemiological studies. Future regional work should also examine household clustering, reinfection patterns, adherence barriers, and care pathways to strengthen external validity and inform context-specific implementation.

## 4 Conclusion

Enterobiasis remains a common, distressing, and eminently controllable infection. The recommendation for two-dose anthelmintic therapy administered 14 days apart is supported by moderate to high-certainty evidence, including pooled analyses and consistent international guideline recommendations. Apparent differences between agents (mebendazole, albendazole, pyrantel) are small and clinically overshadowed by implementation factors, particularly adherence to the second dose and synchronized household treatment. Tailored approaches during pregnancy and early infancy, plus coordination with schools and public-health teams during clusters, complete a rigorous, family-centered strategy.
